# Use of synergistic mixture of chelating agents for *in situ* LDH growth on the surface of PEO-treated AZ91

**DOI:** 10.1038/s41598-020-65396-0

**Published:** 2020-05-26

**Authors:** E. Petrova, M. Serdechnova, T. Shulha, S. V. Lamaka, D. C. F. Wieland, P. Karlova, C. Blawert, M. Starykevich, M. L. Zheludkevich

**Affiliations:** 1 Faculty of Chemistry, Belarusian State University, Nezavisimosti Avenue 4, 220030 Minsk, Belarus; 2 MagIC-Magnesium Innovation Center, Helmholtz-Zentrum Geesthacht, Max-Planck-Straβe 1, 21502 Geesthacht, Germany; 3 Department of Materials and Ceramic Engineering, CICECO - Aveiro Institute of Materials, University of Aveiro, 3810-193 Aveiro, Portugal; 4 Faculty of Engineering, University of Kiel, Kaiserstraße 2, 24143 Kiel, Germany

**Keywords:** Chemical engineering, Inorganic chemistry, Chemical synthesis

## Abstract

The principal possibility to grow layered double hydroxide (LDH) at ambient pressure on plasma electrolytic oxidation (PEO) treated magnesium alloy AZ91 in the presence of chelating agents is demonstrated for the first time. It avoids hydrothermal autoclave conditions, which strongly limit wide industrial application of such coating systems, and the presence of carbonate ions in the electrolyte, which lead to the formation of “passive” non-functionalizable LDH. A combination of chelating agents (sodium diethylenetriamine-pentaacetate (DTPA) and salicylate) were introduced to the treatment solution. The role of each additive and the influence of treatment bath composition on the LDH formation processes are discussed. A synergistic effect of DTPA and salicylate during LDH formation is discovered and its possible explanation is proposed.

## Introduction

Plasma electrolytic oxidation (PEO) treatment is an effective way to improve corrosion and wear resistance of Mg alloys due to the formation of ceramic oxide layers^1–6^. The main advantages of PEO treatment as compared to classical anodization are related to the higher current and voltage applied, introducing micro-discharges, thus changing phase composition, increasing layer thickness and improving properties. Moreover, PEO treatments are normally performed in diluted alkaline electrolytes^7,8^. Thus, the process is significantly more environmentally friendly in comparison with conventional chromic acid anodizing, previously widely used for corrosion protection and forbidden now due to the high toxicity for humans and for environment^9^.

In previous publications it was shown, that corrosion behavior and wear resistance of PEO-coated magnesium alloys depend strongly on the treatment conditions, such as voltage, current density, treatment time and electrolyte composition^2,10–12^. However, since micro-discharges are responsible for coating formation, the produced PEO coatings always contain a number of discharge channels, pores and micro-cracks. These defects strongly compromise the corrosion resistance of PEO layers, because the corrosive solution can easily penetrate through them directly to the metallic interface. Thus, sealing the pores is an effective way to improve long-term corrosion resistance of PEO-treated magnesium alloys. Conventional sealing approaches traditionally include application of organic polymers^13,14^ and compounds^15^ and/or different post-treatment in electrolytes containing lanthanum^16^, silane^17^ or cerium^14,18,19^.

In the last years a lot of attention has been paid to self-healing coatings, which do not only act as barrier layer between the metal and corrosive media, but also release corrosion inhibitors in the case of mechanical damage of the coating. For this purpose, layered double hydroxides are promising materials as they can be effectively intercalated with suitable corrosion inhibiting anions^20–23^. It was shown, that LDH films can be successfully loaded for example with vanadate^24–26^, molybdate^27^ or mercaptobenzothiazolate anions^28,29^ via anionic exchange processes. Up to now, a number of studies demonstrated the possibility of sealing PEO pores on aluminum alloys by *in situ* hydrothermally grown LDH nanocontainers under ambient pressure conditions. In these studies LDHs are produced via one-step processes at 95 °C in an aqueous electrolyte with a substrate acting as a source of Al^3+^ ions through the open PEO pores^30–32^. However, the LDH growth on PEO treated magnesium alloys is not so simple due to the complicity of the system, even if noticeable effect for corrosion protection is expected^33,6^.

Recently, *Zeng et al*. suggested a two-step synthesis of Zn,Al-LDH coating on anodized AZ31 alloy^34^. In that work the preliminary synthesized LDH containers were deposited on the surface of PEO layer by immersion AZ31 sample in the LDH containing solution at autoclave conditions and further covered with poly(lactic acid) coating (PLA). An overall significant improvement of corrosion properties was observed for both LDH and LDH/PLA covered samples in comparison with bare AZ31. *Wu et al*. proposed direct hydrothermal synthesis of Mg,M-LDH films (M = Al, Cr, Fe) on anodized AZ31 with the oxide layer acting as the source of magnesium^35,36^. The same approach was used by *Zhang et al*. for anodized and PEO-treated AZ31 samples^37–39^. In brief, these existing single-step approaches for LDH sealing of oxidized magnesium alloys are performed in autoclaves since they require high pressure conditions and temperatures above 100 °C, which significantly limits the possibility of industrial applications of those methods, e.g. for transport applications. In the cases, when autoclave conditions are not required, LDH formation takes place in carbonated electrolytes^40,41^ and CO_2_ containing environment due to high sorption ability of LDH towards CO_2_^42^. These LDH are extremely hard to functionalize due to the high charge density of carbonate species^43,44^. Thus, formation of “dead” LDH occurs and corrosion inhibitors cannot be intercalated for further “smart” active protection. Overall, LDH sealing of PEO layers is significantly more problematic in the case of magnesium in comparison with aluminum alloys.

Recently, *Shulha et al*. have demonstrated the possibility of Mg,Al-LDH formation on bare AZ91 magnesium alloy via application of chelating agents^45^. Introduction of chelating agents to the reaction system led to the formation of soluble metal complexes and therefore to the increase of concentration of soluble Al(III) and Mg(II) species in the pH range of 9.6–10.3, which is favourable for LDH growth. In this work, sodium salts of salicylic, ethylenediaminetetraacetic and nitrilotriacetic acids were chosen with different complex stability constants with magnesium (log K_Mg-L_) of 4.7, 8.64 and 10.2, respectively. It was shown, that in the solutions containing chelating agents the concentration of soluble forms of magnesium (as Mg^2+^ ions and Mg^2+^-ligand complexes) was maintained relatively high in the pH range necessary for LDH formation (ca. 50% of free Mg^2+^ in the case of nitrilotriacetic acid (NTA) chelating agent addition). No external source of magnesium ions was added to the electrolyte. Instead, chelating agents assisted the dissolution of the substrate, providing enough soluble magnesium species for LDH formation. This approach allowed the formation of LDH flakes on the surface under relatively mild conditions (95 °C and atmospheric pressure) without using carbonate-rich solution. However, an important drawback is the presence of nitrilotriacetic acid as chelating agent. NTA is widely used in cleaning products, because it is easily biodegradable and is almost completely removed during wastewater treatment^46^, but it is considered as carcinogenic^47^, which could cause problems for further industrialization of the process.

In the current study, we demonstrate the possibility of using environmentally friendly chelating agents for direct hydrothermal synthesis of Mg,Al-LDH on the surface and in the pores of PEO-treated AZ91 alloy at 95 °C without employing autoclave conditions. Salicylic acid (SA) and sodium diethylenetriamine-pentaacetate (DTPA) accelerate dissolution of magnesium^48^ as previously shown and are chosen here as chelating agents. A synergistic effect of the combination of DTPA and SA on the processes of LDH formation is discovered and their role in LDH formation is studied.

## Materials

The following materials were used for PEO synthesis: potassium hydroxide (KOH, ≥ 85%, ChemSolute, Germany), sodium phosphate (Na_3_PO_4_, ≥96.0%, pure, anhydrous, ACROS Organics, Germany), sodium aluminate (NaAlO_2,_ (Al_2_O_3_ 50-56%, Na_2_O 40-45%, Fe_2_O_3_ ≤ 0.05%), anhydrous, techn., Sigma-Aldrich Laborchemikalien GmbH, Germany). Magnesium alloy AZ91 was used as a substrate for PEO processing followed by LDH growth. The nominal composition [in wt. %] was: 8.60 Al, 0.64 Zn, 0.22 Mn, 0.027 Nd, 0.0073 Si, 0.0053 Sn, 0.0046 La, 0.0023 Cu, 0.0010 Fe, 0.0003 Ni, 0.00076 Be, 0.0007 Ti, <0.02 Th, <0.0009 Ce, <0.0006 Zr, <0.0004 Pb, <0.0002 Pr, <0.0001 Ag and Mg balance.

The materials used for LDH growth on PEO treated AZ91 alloy are: aluminum nitrate nonahydrate (Al(NO_3_)_3_ ∙ 9H_2_O, ≥ 98%, CarlRoth GmbH, Germany), sodium nitrate (NaNO_3_, ≥99.0%, Merk KGaA, Germany), sodium hydroxide (NaOH, ≥99%, Merk KGaA, Germany), salicylic acid (C_7_H_6_O_3_, ≥99%, Sigma-Aldrich Chemie GmbH, Germany), diethylenetriamine-pentaacetic acid pentasodium salt solution (DTPA, C_14_H_18_N_3_O_10_Na_5_, ~40% in H_2_O, Sigma-Aldrich Chemie GmbH, Germany). AZ91 flakes without PEO coating were used as an additional source of magnesium. Deionized water was used as a solvent.

### Methods

#### PEO preparation

First, AZ91 alloy coupons with a size of 15 × 15 × 4 mm were ground with SiC paper up to 1200 grit, rinsed with deionised water and dried with a stream of cold air. PEO processing of the specimen was conducted for 11 min with a bipolar power supply pe861-UA 500-10-24-S (plating electronic, Sexau, Germany) under the following conditions: current control mode with positive pulses of 3 A, pulse ratio of t_on_: t_off_ = 1 ms: 9 ms. The resulting voltage at the end of the PEO treatment was ~460 V. The electrolyte contained 1 g/L KOH, 8 g/L Na_3_PO_4_ and 12 g/L NaAlO_2_ dissolved in deionized water. The specimen was put into the electrolyte bath with constant stirring and kept at 20 ± 2 °C by a water cooling system. The counter-electrode was made of stainless steel. Finally, the specimens were rinsed in deionized water and dried in warm air.

#### LDH synthesis

Mg,Al-LDH coatings were produced by immersing the specimen for 8 h at 95 °C in 150 ml of solution containing 0.05 M Al(NO_3_)_3_, 0.5 M NaNO_3,_ 0.5 g of AZ91 flakes as additional source of magnesium and aluminum and respective chelating agents: either individual 0.05 M DTPA and 0.003 M SA or both SA + DTPA of the same concentrations. The corresponding samples are referred below as **PEO-D, PEO-S and PEO-DS**, respectively. The initial pH of the solutions was adjusted to 10.0 ± 0.1. After the immersion treatment, the samples were cooled to room temperature and rinsed for 2 min with deionized water under ultrasonication.

### Techniques

#### Thermodynamic calculation

Thermodynamic calculations of the equilibrium composition of the reaction bath were made using Hydra-Medusa software^49^ in order to estimate formation of Mg^2+^ and Al^3+^ complexes in presence of DTPA. The following concentrations were used for calculations: C_14_H_18_N_3_O_10_^5–^ – ﻿0.05 M, Al(NO_3_)_3_ – 0.05 M, NaNO_3_ – 0.5 M, PO_4_^3-^– 0.001 M, Mg^2+^ – 0.1 M (concentration of Mg^2+^ is presented based on the previous work^50^).

#### Characterization

Crystallographic structure of the coatings was studied using Bruker D8 Advance diffractometer (Karlsruhe, Germany) with Cu Kα radiation. The scans were performed in the range of 2 Theta from 5 to 80° (exposure time 1 s, step 0.02°). High resolution two dimensional XRD maps were acquired at the nanofocus end station of the beamline P03 at PETRAIII storage ring (DESY, Hamburg, Germany)^51^. An Eiger 9 M (pixel size 75 µm by 75 µm) and beam size of 1.5 by 1.5 µm was used. The scanning step was 2 µm in vertical direction and 4 µm in horizontal direction, with an acquisition time of 0.5 sec for each point for a XRD pattern. The measured area was 80×80 µm. The energy of the X-rays was 19.7 keV. The horizontal axis of the diffraction patterns were recalculated to Cu K-alpha radiation.

Scanning electron microscope (Vega 3, Tescan, Brno, Czech Republic) equipped with energy dispersive X-ray (EDS) spectrometer (EDS, Heidenrod, Germany) was used to evaluate the surface morphology, cross-sections and composition of the coatings. The cross-sections were prepared using standard metallographic techniques. Glow discharge optical emission spectroscopy (GDOES) was used to investigate the concentration depth profile of the coatings (GD-Profiler 2, HORIBA, Longjumeau, France) with an anode of 4 mm in diameter, at an Ar pressure of 650 Pa and 30 W power.

## Results and Discussion

**Figure 1:**
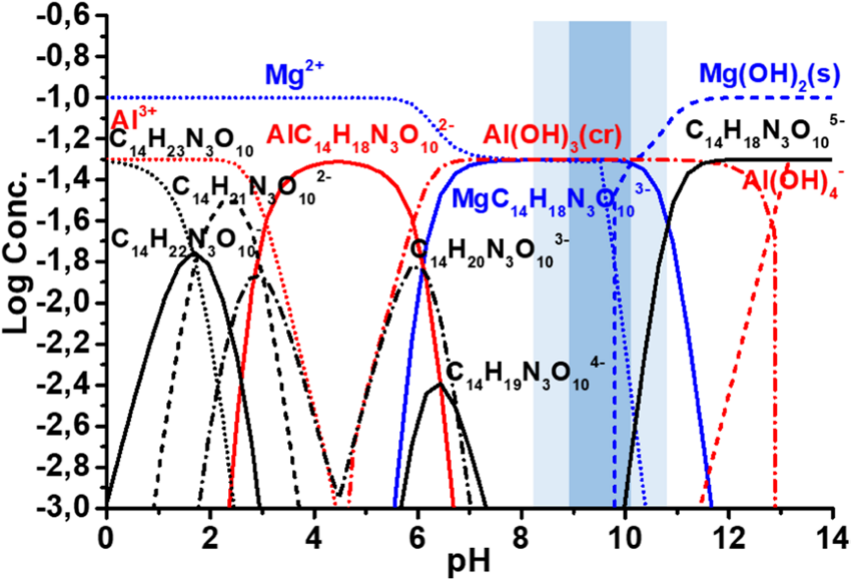
Thermodynamic calculation of the equilibrium composition of different forms of Mg and Al in the solution containing DTPA.

The acceleration effect of different chelating agents on LDH formation on bare AZ91 alloy is related to the increase of soluble Mg^2+^ concentration in the pH range favorable for LDH growth^45^. On the other hand, PEO coatings containing metal oxides can also serve as a potential source of soluble Mg^2+^ species. Previously NTA was found to be the most promising chelating agent for bare AZ91 alloy^45^. In this study, for PEO treated AZ91 DTPA was chosen instead of NTA as an environmentally friendly alternative with similar chelating properties. The selection was done based on thermodynamic calculations performed with Hydra-Medusa software (Fig. 1)^49^. The stability constant of Mg-DTPA complex (7.90-10.70) depends on the ionic strength of the electrolyte^52,53^. In the frame of current work, a value of 9.03 was used as stability constant^54^, which is close to that of NTA (5.50 and 10.20 for ML and ML_2_ complexes, respectively).

Moreover, at pH range 9-11 suitable for LDH formation^28^, following could be observed:

1. Aluminium is present in the same form as it was predicted for successful LDH formation. Its solubility is not differ from one in the presence of NTA^45^.
2. ca. 50% of free magnesium cations are present in the range of possible LDH formation (pH range from 9 till 11 indicated in Fig. 1) and available for the reaction.
3. Phosphates containing in PEO do not show a significant effect on the concentration of Al^3+^ and Mg^2+^ in the pH range suitable for LDH formation (indicated in Fig. 1).

As a result, the conditions for Mg,Al-LDH growth are satisfied: the required concentration of aluminum and magnesium cations are reached in the preferential pH range of LDH formation (pH 9–10)^26^.

**Figure 2:**
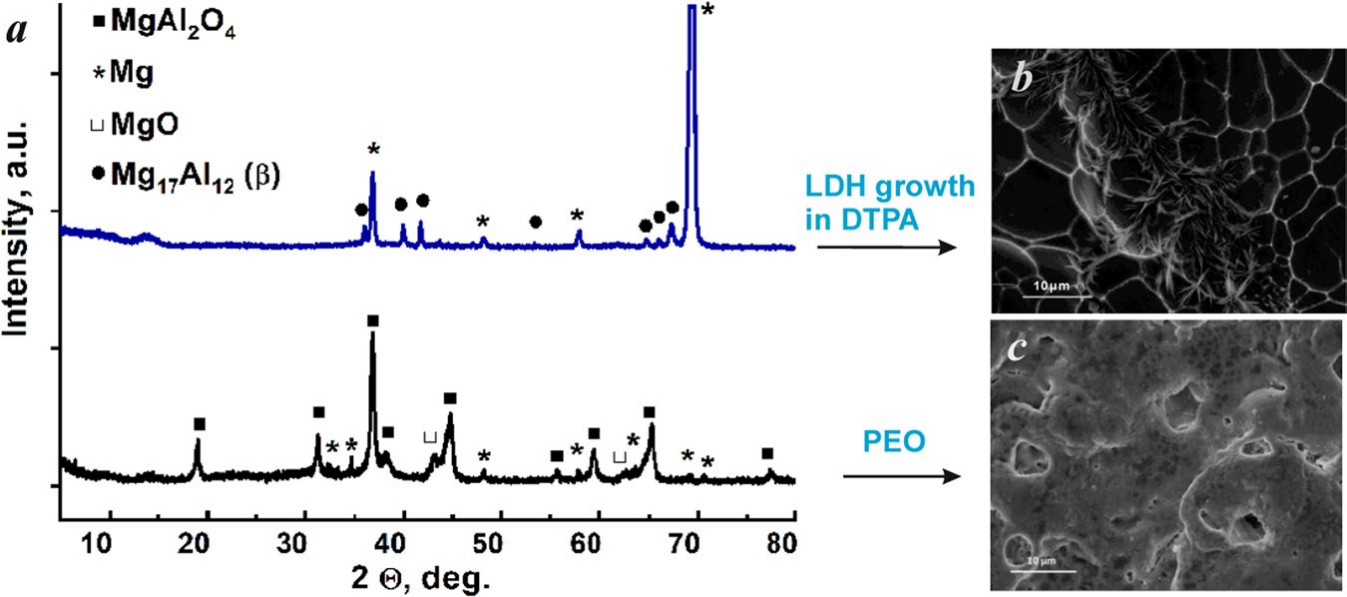
XRD patterns (**a**) and SEM images (**b**,**c**) of the as-prepared PEO sample before and after treatment with a solution of 0.05 M Al(NO_3_)_3_ + 0.5 M NaNO_3_ + 0.05 M DTPA without AZ91 flakes; (3 h, 95 °C, pH = 10.0 ± 0.1).

However, the direct replication of LDH synthesis method on PEO treated AZ91 in the presence of DTPA as chelating agent was not successful – PEO layer was fully etched (Fig. 2b). This observation is confirmed by XRD results (Fig. 2a) – MgAl_2_O_4_ spinel, forming PEO layer on magnesium alloy, could not be detected anymore after the hydrothermal treatment in the solution, containing DTPA. One can also see, that typical pores PEO structure can not be identified via SEM imaging. Instead of it, the grains of bare magnesium alloy are visible.

Different approaches were used in order to influence the kinetics of PEO dissolution and LDH formation:

1. Temperature reduction (from 95 °C to 70 °C),
2. Shortening of treatment time (from 8 h to 0.5 h)
3. Concentration of DTPA species (range from 0.005 M to 0.1 M was investigated).
4. Competitive reactions (external source of magnesium to avoid PEO dissolution and addition of salicylate to prevent fast etching by DTPA).

It was noticed that in the case of PEO-treated AZ91 and in the absence of external Mg source, a complete etching of the PEO layer in the DTPA-containing solution took place in any case (regardless of temperature, DTPA concentration and treatment time). The typical XRD patterns of initial PEO sample before and after treatment with DTPA solution containing only additional Al(III) species are shown in Fig. 2a.

According to XRD results, the PEO coating consists mainly of MgAl_2_O_4_ spinel. This result is in agreement with previously published data for Mg-PEO systems formed in aluminate electrolytes^55–57^. However, the respective XRD peaks of PEO layer, which correspond to MgAl_2_O_4_, disappear completely after 3 h of treatment in a DTPA containing bath, while no LDH is formed yet. No respective diffraction peak is visible in the range of 9.5–11.5°, typical for (003) LDH peak. One can conclude that, on the one hand, the concentration of Mg^2+^ required for LDH formation is not high enough (since the additional source of magnesium helped to overcome the problem) and, on the other hand, that the PEO layer needs to be protected against etching in the presence of such complexants. On the first view, these requirements are contradicting, but a new concept was developed to overcome this problem:

1. 0.5 g of AZ91 flakes were added to the reactive electrolyte as an additional source of magnesium;
2. Salicylate was used as it forms adsorption layer on PEO surface significantly slowing down etching effect characteristic for DTPA^58^.
3. Use of mixture of salicylic acid and DTPA as additives, with an assumption, that DTPA acts as strong Al^3+^ and Mg^2+^ complexing agent hence promoting LDH formation, while SA readily adsorbs on PEO surface and weakens its etching by DTPA. It is known that the dissolution rate of metal (hydr)oxides can be reduced in presence of several complexing agents, even though each of them individually promotes the dissolution due to the formation of surface metal-chelate complexes and surface screening due to adsorption^59–62^.

**Figure 3:**
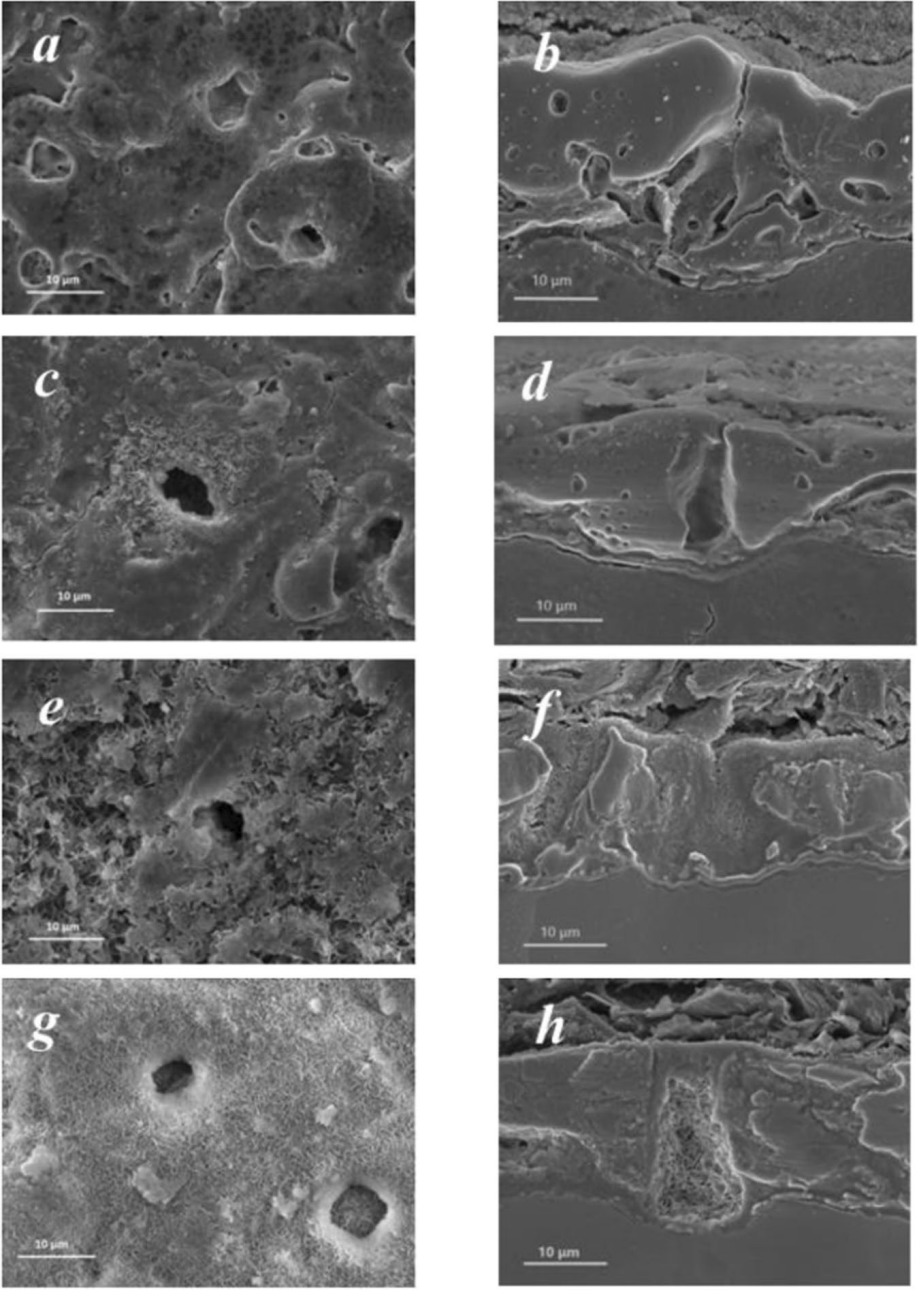
SEM images of PEO-treated AZ91 samples before (**a**,**b**) and after 8 h treatment with AZ91 flakes, and 0.05 M Al(NO_3_)_3_ + 0.5 M NaNO_3_ and different chelating agents: SA (**c**,**d**); DTPA (**e**,**f**); DTPA + SA (**g**,**h**).

The SEM micrographs (top and cross-section view) of the PEO coatings before and after suggested treatments with chelating agents are shown in Fig. 3. It can be seen, that the concept of synergistic DTPA and SA action is the most effective for LDH formation. For instance, in the case of SA (Fig. 3c,d), only a few LDH-like flakes are formed on the surface, while the pores remain LDH-free. In the presence of both DTPA and AZ91 flakes, the sealing of PEO pores with Mg,Al-LDH occurs. However, there is a considerable difference in the morphology of **PEO-D** and **PEO-DS** samples. In the presence of additional salicylate, the topography of LDH-covered sample resembles initial PEO coating, while DTPA without SA leads to deterioration of the top PEO layer (partial etching). Without salicylate present, PEO layer is strongly deteriorated by DTPA, however, some of it still remains on the surface of the alloy. This can be explained by the presence of AZ91 flakes, which act as additional source of Mg(II). In the case when no AZ91 flakes were present in the treatment bath, complete etching of PEO layer took place after 3 h of treatment (Fig. 2). In presence of AZ91 flakes and DTPA, relatively separated LDH flakes are formed on the PEO layer (Fig. 3e,f).

Finally, in the presence of both DTPA and SA the topography of LDH-covered sample resembles initial PEO coating with homogeneous and continuous flake-film (Fig. 3g,h). Therefore SA, although it does not cause a substantial LDH formation, has a significant effect on the structure and morphology of the resulting coatings.

**Figure 4:**
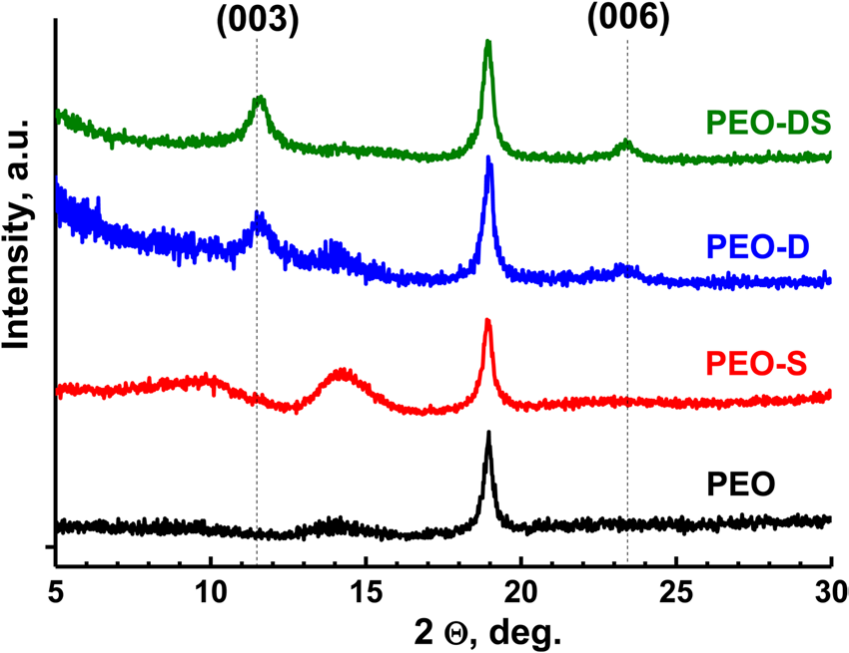
XRD patterns of the PEO samples before and after treatment with a solution containing different chelating agents, AZ91 flakes and 0.05 M Al(NO_3_)_3_ + 0.5 M NaNO_3_; (8 h, 95 °C, pH = 10.0 ± 0.1).

XRD patterns of PEO samples which underwent the LDH treatment in the presence of different chelating agents and AZ91 flakes are shown in Fig. 4. It can be seen, that the spinel part (characteristic peak of MgAl_2_O_4_ is visible at 18.9°) of the initial PEO structure remains intact for all the investigated samples and the formation of Mg,Al-LDH phase took place for **PEO-D** and **PEO-DS** samples. The characteristic peak at 11.5° corresponding to (003) reflection of LDH appears in the case of **PEO-D** and **PEO-DS** samples. This corresponds well with the results previously published^45^ and observed during SEM analysis (Fig. 3), confirming that salicylate is unlikely to promote LDH formation on AZ91-based systems (Fig. 4**, PEO-S sample**). However, simultaneous introduction of both DTPA and SA to the treatment bath positively influences LDH formation. In the case of **PEO-DS** sample, second LDH peak (006 reflection) at 23.3° is clearly visible and the relative intensity of both LDH peaks ((003) and (006) reflections) against spinel PEO peak increases as compared to **PEO-D** sample. These peaks are related to hydroxide-loaded LDH, which is quite typical for LDH grown on various magnesium alloys^45,63,64^. The broad peak around 14° at PEO and PEO-S patterns corresponds to thin nanocrystalline surface film on PEO layer and is not related to LDH phase.

**Figure 5:**
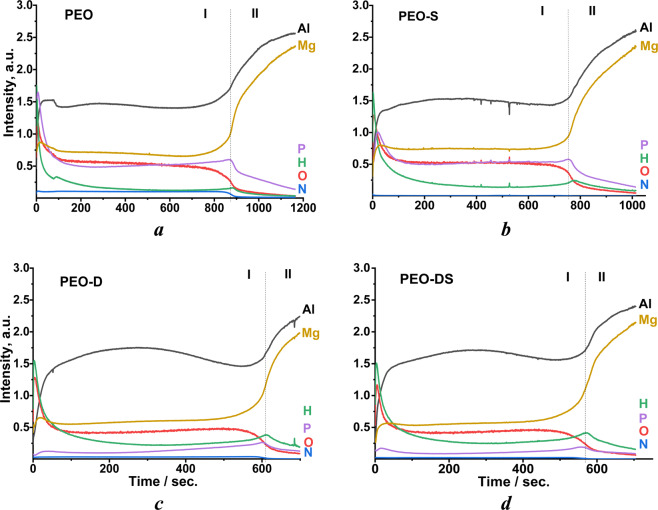
Element depth profiles for initial PEO (**a**) and the samples after treatment with different chelating agents, AZ91 flakes and 0.05 M Al(NO_3_)_3_ + 0.5 M NaNO_3_: PEO-S (**b**); PEO-D (**c**); PEO-DS (**d**) under following conditions: (8 h, 95 °C, pH = 10.0 ± 0.1).

The hybrid PEO/LDH coating system, formed as a result, was studied with GDOES in order to confirm that LDH is formed in the pores of PEO coating and not on the surface only. Depth profile analyses for the main elements across the coating for all the investigate samples are shown in Fig. 5

Overall, two main regions (zones I and II) were found on depth profiles of PEO coatings. The first zone with a relatively high content of aluminum, magnesium, oxygen and phosphorus corresponds to the PEO layer. The second zone is characterized by a higher level of aluminum and magnesium and corresponds to the AZ91 substrate. The thickness of PEO layer decreases after treatment with DTPA-containing solutions, which confirms partial dissolution of the PEO coating due to the etching by DTPA chelating agent.

In contrast to the initial PEO, **PEO-D** and **PEO-DS** samples are characterized by relative increased hydrogen signal throughout the PEO layer. This can be associated with LDH formation (source of hydrogen) not only on the surface, but also inside the PEO pores and their sealing. In the case of **PEO-DS** sample, the relative intensity of hydrogen signal is even higher. This corresponds well with the XRD data and proves that addition of SA increases the amount of formed LDH. In contrast, for the **PEO-S** sample almost no increase in the hydrogen signal is observed, confirming that there is no LDH formed inside of PEO pores. Unfortunately, based on GDOES results it is not possible to judge, which compounds (organic chelating agents, inorganic LDH species, etc.) are exactly responsible for this hydrogen signal. In order to understand the mechanism of LDH formation on PEO treated magnesium alloys in detail, additional investigations will be required.

Another characteristic feature of **PEO-D** and **PEO-DS** samples in comparison with parental PEO is a significant decrease of phosphorus and magnesium signals within PEO layer, while Al signal remains at the same level. This can be explained by preferential interaction of DTPA with phosphorus-rich part of PEO layer (most likely amorphous Mg_3_(PO_4_)_2_, which is not visible in XRD patterns, but was detected in similar coatings^65,66^) rather than with spinel phase. However, the reaction between DTPA and spinel part of PEO takes place as well, which can be seen from the aluminum distribution profile of the **PEO-D** sample. The intensity of Al signal decreases at the beginning and at end of zone I which represents PEO layer. Therefore, a conclusion can be made, that DTPA penetrates the pores and promotes the dissolution of the inner (barrier) layer of the coating as well as the surface. This suggestion is in a good agreement with our first results, when no PEO layer was seen on the magnesium surface after LDH synthesis without addition of AZ91 flakes and salicylic acid in the solution: the flaking off of the coating was strongly supported by destruction of inner PEO layer.

This process, however, might be inhibited by protective effect of salicylate. It can be seen via the absence of such or much weaker decrease of aluminum signal in presence of SA (**PEO-S** and **PEO-DS** samples) in the GDOES elemental distribution profiles. In other words, the introduction of salicylate to the reaction bath changes the mechanism of PEO layer etching and/or changes the preferentially soluble components from the layer. This is likely due to the adsorption of salicylate on the bottom of PEO pores preventing or reducing the destruction of barrier layer by DTPA.

**Figure 6:**
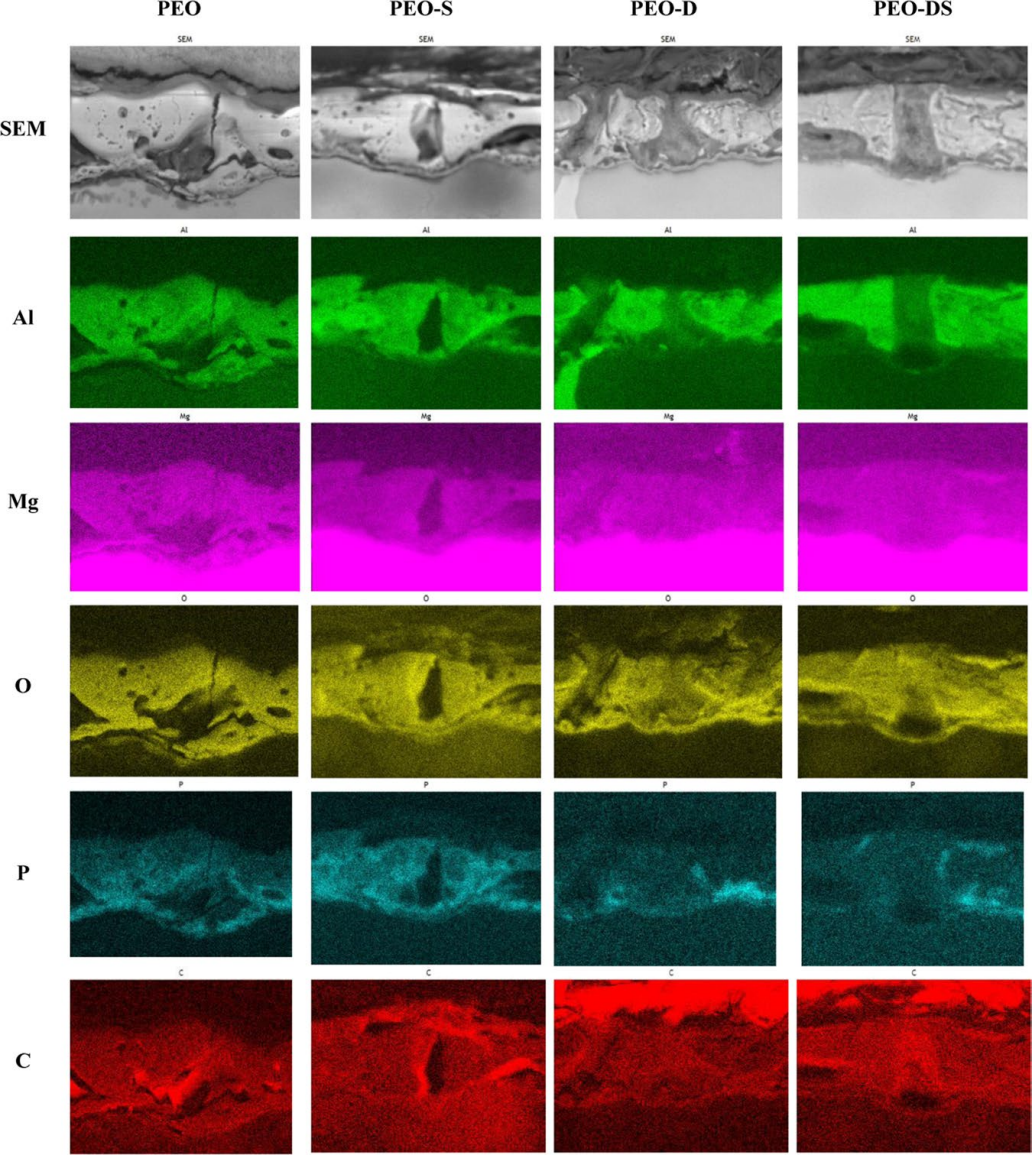
Distribution of the relevant elements of PEO-treated AZ91 samples before and after 8 h of treatment with different chelating agents.

Overall, the mechanism of PEO–chelating agent interaction and subsequent LDH formation is yet not fully understood. According to GDOES data, the content of phosphorus in the PEO layer decreases after treatment with DTPA containing solutions. This was additionally confirmed by EDX mapping to determine the elemental distribution in the coatings (Fig. 6). It can be seen, that spinel part of the coating, which contains magnesium-aluminum oxide, remains almost intact after DTPA and SA treatment. This result is in good agreement with XRD measurements (Fig. 4, spinel peak at 18.9°). As demonstrated by GDOES data, aluminum containing part of PEO layer tends to dissolve from the bottom of pores as a result of interaction with DTPA, which does not happen in presence of salicylate. This can also be seen from EDX mapping: in the case of parental PEO and the samples treated in the presence of SA, thin layer with high concentration of aluminum is located at the bottom of a pore. In contrast, for **PEO-D** sample the pore bottom is depleted of aluminum. This might be due to adsorption of SA on the surface of oxide layer. In the presence of SA, the concentration of Al-DTPA complexes on the surface of PEO layer should decrease, and the dissolution of spinel slows down.

However, the phosphorus containing part of the coating almost disappears as a result of interaction with DTPA. The introduction of salicylate changes this behavior and therefore keeps PEO layer almost intact. Most likely it also preserves its mechanical and barrier properties after sealing with LDH, but this will be investigated additionally in future studies.

**Figure 7:**
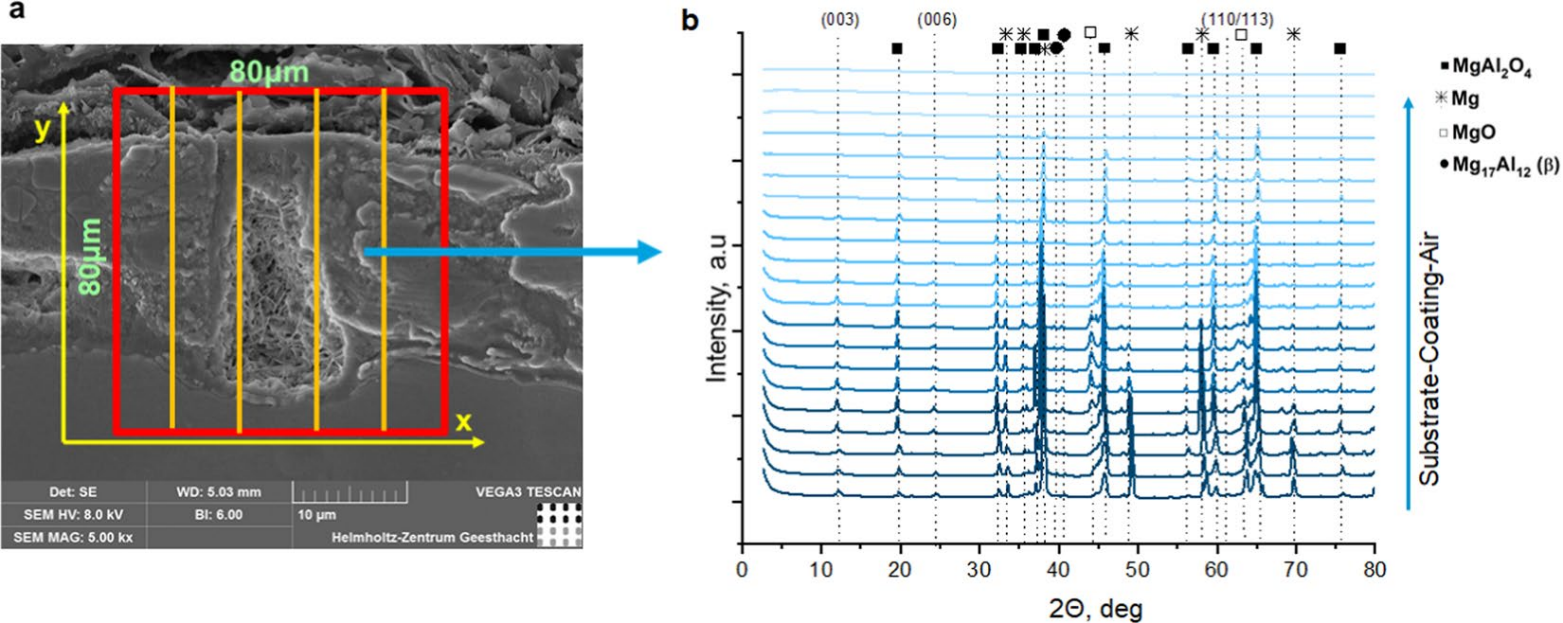
Synchrotron XRD scanning scheme (**a**) and XRD patterns of one line in Y-direction (**b**) in case of **PEO-DS** sample (presented for clarity).

**Figure 8:**
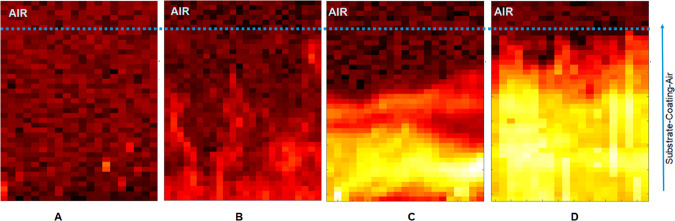
2D maps of LDH distribution of PEO-treated AZ91 samples with different chelating agents: PEO (**A**), PEO-S (**B**), PEO-D (**C**), PEO-DS (**D**). The scaling in the (**A)** to D is adjusted, allowing a direct comparison.

Synchrotron XRD measurements were conducted to investigate the LDH distribution in pores of PEO coatings. For this a two-dimensional maps of the sample at the air/PEO/substrate interface were created for each sample (Fig. 7). From the diffraction patterns, the intensity of the (003) LDH peak was extracted and a two-dimensional maps showing the spatial intensity variation were composed (Fig. 8). The maps of LDH distribution correspond well with XRD and GDOES data. No LDH phase is present in the case of initial PEO and only trace amounts of it can be seen at the map of **PEO-S** sample (Fig. 8A,B, respectively). In contrast, for **PEO-D** and **PEO-DS** samples the formation of LDH phase throughout the PEO coating is confirmed (Fig. 8C,D). In the case of the **PEO-D** sample LDH is formed within the PEO layer, and the intensity of the LDH signal increases closer to AZ91 interface. This is confirmed by GDOES data on partial dissolution of inner layer of PEO coating (Fig. 5c), which is likely to make it less stable with a tendency to flake off. The relative intensity of LDH signal for **PEO-DS** sample is higher, which proves that more LDH is formed in the presence of both DTPA and SA.

Overall, as the next step, a deeper investigation of the mechanisms of PEO interaction with different chelating agents and the optimization of LDH sealing conditions will be conducted. Furthermore, the functionalization of formed LDH nanocontainers with different species (e.g. corrosion inhibitors) and testing of formed hybrid systems for possible industrial applications are necessary. Beyond the frame of this work, we got a preliminary confirmation that this approach can also be extended for other magnesium-based substrates, such as PEO-treated AZ31 alloy.

## Conclusion

The principal possibility of LDH growth on PEO-treated magnesium alloy AZ91 under ambient pressure is demonstrated in this work. In order to avoid the hydrothermal autoclave conditions, organic additives (DTPA and SA) were successfully added to the treatment bath. It was shown that sealing of PEO pores with LDH nanocontainers is possible. The suggested mechanism includes formation of Me-ligand complexes during DTPA-assisted partial dissolution of PEO layer. A synergistic effect of SA addition on the surface morphology of complex PEO-LDH coating was discovered. It was explained via the formation of salicylate adsorption layer on PEO surface that hampers access of DTPA to PEO surface and weakens its dissolution. The results of this work are promising for further development of LDH-sealed porous coatings on magnesium alloys, their further functionalization and industrial application.

## References

[1] Heimann Robert B., Lehmann Hans D. (2015). Bioceramic Coatings for Medical Implants.

[2] Pezzato L (2018). Tribocorrosion Properties of PEO Coatings Produced on AZ91 Magnesium Alloy with Silicate- or Phosphate-Based Electrolytes. Coatings..

[3] Blawert C, Dietzel W, Ghali E, Song G (2006). Anodizing Treatments for Magnesium Alloys and Their Effect on Corrosion Resistance in Various Environments. Advanced Engineering Materials.

[4] Krishna LR, Purnima AS, Sundararajan G (2006). A comparative study of tribological behavior of microarc oxidation and hard-anodized coatings. Wear..

[5] Pezzato L, Brunelli K, Napolitani E, Magrini M, Dabalà M (2015). Surface properties of AZ91 magnesium alloy after PEO treatment using molybdate salts and low current densities. Applied Surface Science.

[6] Song YL, Liu YH, Yu SR, Zhu XY, Wang Q (2008). Plasma electrolytic oxidation coating on AZ91 magnesium alloy modified by neodymium and its corrosion resistance. Applied Surface Science.

[7] Guo HF, An MZ, Huo HB, Xu S, Wu LJ (2006). Microstructure characteristic of ceramic coatings fabricated on magnesium alloys by micro-arc oxidation in alkaline silicate solutions. Applied Surface Science.

[8] Rama Krishna L, Sundararajan G (2014). Aqueous Corrosion Behavior of Micro Arc Oxidation (MAO)-Coated Magnesium Alloys: A Critical Review. JOM..

[9] Acids generated from chromium trioxide and their oligomers. - Authorisation list - ECHA., Echa.Europa.Eu. (2019). https://echa.europa.eu/authorisation-list/-/dislist/details/0b0236e1807dfaad, accessed July 23, (2019).

[10] Pezzato L, Brunelli K, Gross S, Magrini M, Dabalà M (2014). Effect of process parameters of plasma electrolytic oxidation on microstructure and corrosion properties of magnesium alloys. Journal of Applied Electrochemistry.

[11] Lu X (2018). Influence of particle additions on corrosion and wear resistance of plasma electrolytic oxidation coatings on Mg alloy. Surface and Coatings Technology.

[12] Ardelean H, Frateur I, Zanna S, Atrens A, Marcus P (2009). Corrosion protection of AZ91 magnesium alloy by anodizing in niobium and zirconium-containing electrolytes. Corrosion Science..

[13] Yang J (2018). Enhanced Wear Performance of Hybrid Epoxy-Ceramic Coatings on Magnesium Substrates. ACS Applied Materials & Interfaces.

[14] Mingo B (2018). Influence of sealing post-treatments on the corrosion resistance of PEO coated AZ91 magnesium alloy. Applied Surface Science.

[15] Chirkunov, A. A. *et al.* Corrosion protection of magnesium alloy by PEO-coatings containing sodium oleate, *Int. J. Corros. Scale Inhib.* **8,** 1170–1188, 10.17675/2305-6894-2019-8-4-22 (2019).

[16] Pezzato Luca, Brunelli Katya, Babbolin Riccardo, Dolcet Paolo, Dabalà Manuele (2017). Sealing of PEO Coated AZ91 Magnesium Alloy Using La-Based Solutions. International Journal of Corrosion.

[17] Wang YQ, Deng YZ, Shao YW, Wang FH (2014). New sealing treatment of microarc oxidation coating. Surface Engineering..

[18] Phuong NV, Fazal BR, Moon S (2017). Cerium- and phosphate-based sealing treatments of PEO coated AZ31 Mg alloy. Surface and Coatings Technology.

[19] Mohedano M, Blawert C, Zheludkevich ML (2015). Cerium-based sealing of PEO coated AM50 magnesium alloy. Surface and Coatings Technology.

[20] Hang TTX, Truc TA, Duong NT, Pébère N, Olivier M-G (2012). Layered double hydroxides as containers of inhibitors in organic coatings for corrosion protection of carbon steel. Progress in Organic Coatings.

[21] Hang To Thi Xuan, Truc Trinh Anh, Duong Nguyen Thuy, Vu Pham Gia, Hoang Thai (2012). Preparation and characterization of nanocontainers of corrosion inhibitor based on layered double hydroxides. Applied Clay Science.

[22] Tedim J, Zheludkevich ML, Salak AN, Lisenkov A, Ferreira MGS (2011). Nanostructured LDH-container layer with active protection functionality. Journal of Materials Chemistry.

[23] Guo X, Zhang F, Evans DG, Duan X (2010). Layered double hydroxide films: synthesis, properties and applications. Chem. Commun..

[24] Li Y, Li S, Zhang Y, Yu M, Liu J (2015). Enhanced protective Zn–Al layered double hydroxide film fabricated on anodized 2198 aluminum alloy. Journal of Alloys and Compounds.

[25] Guo L (2018). A comparison of corrosion inhibition of magnesium aluminum and zinc aluminum vanadate intercalated layered double hydroxides on magnesium alloys. Frontiers of Materials Science.

[26] Yasakau KA, Tedim J, Zheludkevich ML, Ferreira MGS (2014). Active Corrosion Protection by Nanoparticles and Conversion Films of Layered Double Hydroxides. CORROSION..

[27] Klemkaitė-Ramanauskė K, Žilinskas A, Taraškevičius R, Khinsky A, Kareiva A (2014). Preparation of Mg/Al layered double hydroxide (LDH) with structurally embedded molybdate ions and application as a catalyst for the synthesis of 2-adamantylidene(phenyl)amine Schiff base. Polyhedron..

[28] Serdechnova M (2016). Interlayer intercalation and arrangement of 2-mercaptobenzothiazolate and 1,2,3-benzotriazolate anions in layered double hydroxides: *In situ* X-ray diffraction study. Journal of Solid State Chemistry.

[29] Poznyak SK (2009). Novel Inorganic Host Layered Double Hydroxides Intercalated with Guest Organic Inhibitors for Anticorrosion Applications. ACS Applied Materials & Interfaces.

[30] Serdechnova M (2017). PEO Coatings with Active Protection Based on *In-Situ* Formed LDH-Nanocontainers. Journal of The Electrochemical Society.

[31] Serdechnova M (2017). Role of Phase Composition of PEO Coatings on AA2024 for *In-Situ* LDH Growth. Coatings..

[32] Mohedano M (2017). Active protective PEO coatings on AA2024: Role of voltage on *in-situ* LDH growth. Materials & Design.

[33] Buling A, Zerrer J (2019). Increasing the application fields of magnesium by ultraceramic: Corrosion and wear protection by plasma electrolytical oxidation (PEO) of Mg alloys. Surface and Coatings Technology.

[34] Zeng R-C (2015). Corrosion resistance of Zn–Al layered double hydroxide/poly(lactic acid) composite coating on magnesium alloy AZ31. Frontiers of Materials Science.

[35] Wu L (2018). Fabrication and characterization of Mg-M layered double hydroxide films on anodized magnesium alloy AZ31. Applied Surface Science.

[36] Wu L (2017). Communication—Fabrication of Protective Layered Double Hydroxide Films by Conversion of Anodic Films on Magnesium Alloy. Journal of The Electrochemical Society.

[37] Zhang G (2018). Effect of Micro-Arc Oxidation Coatings Formed at Different Voltages on the *In Situ* Growth of Layered Double Hydroxides and Their Corrosion Protection. Journal of The Electrochemical Society.

[38] Zhang G (2018). Active corrosion protection by a smart coating based on a MgAl-layered double hydroxide on a cerium-modified plasma electrolytic oxidation coating on Mg alloy AZ31. Corrosion Science..

[39] Zhang G (2018). Sealing of anodized magnesium alloy AZ31 with MgAl layered double hydroxides layers. RSC Advances.

[40] Chen J, Song Y, Shan D, Han E-H (2011). *In situ* growth of Mg–Al hydrotalcite conversion film on AZ31 magnesium alloy. Corrosion Science..

[41] Chen J, Song Y, Shan D, Han E-H (2012). Study of the *in situ* growth mechanism of Mg–Al hydrotalcite conversion film on AZ31 magnesium alloy. Corrosion Science..

[42] Zhao Y (2019). Two-dimensional-related catalytic materials for solar-driven conversion of CO x into valuable chemical feedstocks. Chem. Soc. Rev..

[43] Nakayama H, Hayashi A (2014). Mixing Acid Salts and Layered Double Hydroxides in Nanoscale under Solid Condition. Pharmaceutics..

[44] Miyata S (1983). Anion-Exchange Properties of Hydrotalcite-Like Compounds. Clays and Clay Minerals.

[45] Shulha TN (2018). Chelating agent-assisted *in situ* LDH growth on the surface of magnesium alloy. Scientific Reports.

[46] Nitrilotriacetic acid, En.Wikipedia.Org., (2019), https://en.wikipedia.org/wiki/Nitrilotriacetic_acid (accessed August 1, 2019), (2019).

[47] NTP (National Toxicology Program)., Report on Carcinogens, Fourteenth Edition. Research Triangle Park, NC: U.S. Department of Health and Human Services, Public Health Service, (2016).

[48] Lamaka SV (2017). Comprehensive screening of Mg corrosion inhibitors. Corrosion Science..

[49] I. Puigdomenech, Hydra-Medusa software, Chemical Equilibrium Diagrams. (2017), https://sites.google.com/site/chemdiagr/home (accessed September 23, 2019).

[50] Karavai OV (2010). Localized electrochemical study of corrosion inhibition in microdefects on coated AZ31 magnesium alloy. Electrochimica Acta..

[51] Krywka C (2012). A two-dimensional waveguide beam for X-ray nanodiffraction. Journal of Applied Crystallography.

[52] Crea F, De Stefano C, Gianguzza A, Piazzese D, Sammartano S (2003). Speciation of poly-amino carboxylic compounds in seawater. Chemical Speciation & Bioavailability.

[53] De Stefano C, Gianguzza A, Piazzese D, Sammartano S (2003). Interactions of diethylenetriaminepentaacetic acid (dtpa) and triethylenetetraaminehexaacetic acid (ttha) with major components of natural waters. Analytical and Bioanalytical Chemistry.

[54] Přibil R. (1972). TITRIMETRIC ANALYSIS. Analytical Applications of EDTA and Related Compounds.

[55] R.O. Hussein, X. Nie, D.O. Northwood, Plasma electrolytic oxidation (PEO) coatings on Mg-alloys for improved wear and corrosion resistance, in: València, Spain, 2015: pp. 163–176. 10.2495/SECM150151.

[56] Tu W (2017). Plasma electrolytic oxidation of AZ31 magnesium alloy in aluminate-tungstate electrolytes and the coating formation mechanism. Journal of Alloys and Compounds.

[57] Barati Darband Gh., Aliofkhazraei M., Hamghalam P., Valizade N. (2017). Plasma electrolytic oxidation of magnesium and its alloys: Mechanism, properties and applications. Journal of Magnesium and Alloys.

[58] Maltseva, A. Evolution de surface lors de la corrosion de magnésium: nouvelles approches analytiques pour comprendre les mécanismes de corrosion et de protection, 2018. http://www.theses.fr/s176048 (accessed October 24, 2019).

[59] Kraemer SM, Chiu VQ, Hering JG (1998). Influence of pH and Competitive Adsorption on the Kinetics of Ligand-Promoted Dissolution of Aluminum Oxide. Environmental Science & Technology.

[60] Biber Madeleine V., Stumm Werner. (1994). An In-Situ ATR-FTIR Study: The Surface Coordination of Salicylic Acid on Aluminum and Iron(III) Oxides. Environmental Science & Technology.

[61] Žutić V, Stumm W (1984). Effect of organic acids and fluoride on the dissolution kinetics of hydrous alumina. A model study using the rotating disc electrode. Geochimica et Cosmochimica Acta.

[62] Tombácz E. (2003). Effect of Environmental Relevant Organic Complexants on the Surface Charge and the Interaction of Clay Mineral and Metal Oxide Particles. Role of Interfaces in Environmental Protection.

[63] Ishizaki T, Miyashita T, Inamura M, Nagashima Y, Serizawa A (2019). Effect of Al Content in the Mg-Based Alloys on the Composition and Corrosion Resistance of Composite Hydroxide Films Formed by Steam Coating. Materials..

[64] Wu L, Pan F, Liu Y, Zhang G, Tang A, Atrens A (2018). Influence of pH on the growth behaviour of Mg–Al LDH films. Surface Engineering..

[65] Arrabal R, Matykina E, Viejo F, Skeldon P, Thompson GE (2008). Corrosion resistance of WE43 and AZ91D magnesium alloys with phosphate PEO coatings. Corrosion Science..

[66] Zhuang J, Song R, Li H, Xiang N (2018). Effect of Various Additives on Performance of Plasma Electrolytic Oxidation Coatings Formed on AZ31 Magnesium Alloy in the Phosphate Electrolytes. Journal of Wuhan University of Technology-Mater. Sci. Ed.

